# Long Non-Coding RNA MAFG-AS1 as a Potential Biomarker for Hepatocellular Carcinoma: Linkage with Tumor Features, Markers, Liver Functions, and Survival Profile

**DOI:** 10.3389/fsurg.2022.848831

**Published:** 2022-05-17

**Authors:** Yuanyuan Tian, Jiao Wang, Ge Tian, Bing Li, Moli Chen, Xiaoning Sun

**Affiliations:** ^1^ Department of Gastroenterology, Hainan General Hospital/Hainan Affiliated Hospital of Hainan Medical University, Haikou, China; ^2^ Department of Infectious Diseases, Hainan General Hospital/Hainan Affiliated Hospital of Hainan Medical University, Haikou, China; ^3^ Section of Scientific Research, Beijing Xian Nong Tan Sports Technical College, Beijing, China; ^4^ Clinical College, Hainan Medical University, Haikou, China

**Keywords:** hepatocellular carcinoma, lnc-MAFG-AS1, tumor features, tumor markers, prognosis

## Abstract

**Purpose:**

Long non-coding RNAs musculoaponeurotic fibrosarcoma oncogene family, protein G antisense 1 (lnc-MAFG-AS1) regulates hepatocellular carcinoma (HCC) progression and treatment resistance in multiple ways, while its engagement in HCC clinical management remains obscure. The current study aims to explore the relationship of lnc-MAFG-AS1 with tumor features, liver function indexes, tumor markers, and prognosis in HCC patients.

**Methods:**

One hundred and fifty-two surgical HCC patients who underwent tumor resection were retrospectively analyzed. Their tumor and adjacent tissues were acquired and then proposed to reverse transcription-quantitative polymerase chain reaction to detect lnc-MAFG-AS1 expression.

**Results:**

Lnc-MAFG-AS1 expression was increased in HCC tumor tissue than in adjacent tissue [median (interquartile range): 2.730 (1.685–4.198) vs. 0.990 (0.703–1.468), *p *< 0.001], with a high area under the curve [0.889, 95% confidence interval (CI): 0.854–0.924] to distinguish them via receiver operating characteristic curve analysis. Tumor lnc-MAFG-AS1 was linked with multifocal nodules (*p *< 0.001), increased Barcelona Clinic Liver Cancer (BCLC) stage (*p *= 0.018), and elevated China Liver Cancer (CNLC) stage (*p *= 0.008), which also correlated with an abnormal alpha-fetoprotein (AFP) level (*p *= 0.004), However, lnc-MAFG-AS1 was not linked with other disease conditions, tumor properties, liver function indexes, or tumor markers (all *p*s* *> 0.05). In addition, patients with a high expression of lnc-MAFG-AS1 exhibited worse overall survival than those with a low expression of lnc-MAFG-AS1 [median (95% CI): 34.0 (24.5–43.5) vs. 48.0 (41.5–54.5) months] (*p *= 0.011), which was further validated by univariate Cox’s analysis [hazard ratio (HR) = 1.827, *p *= 0.013] and multivariate Cox’s analysis (HR = 1.697, *p *= 0.040).

**Conclusion:**

Lnc-MAFG-AS1 relates to multifocal nodules, increased BCLC stage, elevated CNLC stage, and abnormal AFP level and predicts pejorative prognosis in HCC patients.

## Introduction

Hepatocellular carcinoma (HCC) ranks as one of the most prevalent and deadly cancers with 906,000 new cases and 830,000 deaths per year globally (1). Several disease-stage classifications are proposed to stratify treatment options and estimate the prognosis of HCC, such as Barcelona Clinic Liver Cancer (BCLC) and China Liver Cancer (CNLC) stages (2, 3). Among the recommended treatment choices for HCC, surgical resection with or without neoadjuvant/adjuvant therapy is still the most convincing (4–6). However, the prognosis still differs among patients receiving tumor resection due to tumor heterogeneity and responsiveness. Therefore, prognostic markers are now emerging, drawing great attention from the medical fraternity.

Long non-coding RNAs musculoaponeurotic fibrosarcoma oncogene family, protein G antisense 1 (lnc-MAFG-AS1), which belongs to non-coding RNAs that lack protein-coding ability with a length of more than 200 bp, has been recently discovered to be an oncogene in several cancers (7–12). For example, lnc-MAFG-AS1 facilitates gastric cancer growth via serving as competing endogenous RNA of microRNA (miR)-505 for polo-like kinase-1 (7); another study has found that lnc-MAFG-AS1 induces ovarian cancer progression by activating NF-κB1-mediated insulin-like growth factor 1 (IGF1) (8). From the perspective of HCC, lnc-MAFG-AS1 is known to increase its proliferation, migration, invasion, and epithelial–mesenchymal transition (EMT) through sponging miR-6852 and miR-3196/OTX1 axis (13, 14). Besides, lnc-MAFG-AS1 enhances drug resistance by regulating miR-3196-mediated STRN4 in HCC (15). Based on the above information, it is hypothesized that lnc-MAFG-AS1 possesses potency as a biomarker in HCC.

Therefore, the current study aims to investigate the relationship of lnc-MAFG-AS1 with tumor features, liver function indexes, tumor markers, and prognosis in surgical HCC patients.

## Methods

### Patients

This study retrospectively analyzed 152 surgical HCC patients treated by surgical resection from the period January 2016 to December 2019. The screening of patients was based on the following criteria: (1) those who had a pathological diagnosis of primary HCC, (2) those primarily treated by surgical resection, (3) those who had retrievable tumor and adjacent tissues that were fresh-frozen in liquid nitrogen, and (4) those whose preoperative clinical data, laboratory test data, as well as survival data were accessible and available. The following patients were omitted: (1) those who had tumor or adjacent tissue specimens were not eligible for RNA isolation due to improper preservation, (2) those who had a history of other cancers, and (3) those who had failed to follow-up within 3 months after surgery. Ethical approval was acquired from the Ethics Committee. Written informed consent was required from either the patients or their families.

### Specimen Acquisition

All patients’ tumor and adjacent tissues (stored in liquid nitrogen) were acquired from the hospital specimen library. All samples were cryopreserved and feasible for reverse transcription-quantitative polymerase chain reaction (RT-qPCR) assay. In addition, patients’ clinical data, including basic information, tumor features, liver function indexes, and tumor markers, were also abstracted from their medical records. The CNLC stage of the patients was retrospectively assessed according to the documented tumor features (16). Moreover, overall survival (OS) time up to June 2021 was calculated on the basis of follow-up records. Apart from surgical resection, 8 patients received neoadjuvant sorafenib, 13 patients received neoadjuvant transarterial chemoembolization (TACE), and 37 patients received adjuvant treatment (such as TACE, chemotherapy, and interferon).

### Lnc-MAFG-AS1 Detection

RT-qPCR was carried out to detect lnc-MAFG-AS1 in the tumor and adjacent tissues. The RT-qPCR procedures were implemented as described in a previous study (17), using the following kits: TRIzol™ Reagent (Invitrogen™, Carlsbad, CA, USA) for total RNA extraction; iScript™ cDNA Synthesis Kit (Bio-Rad, Hercules, CA, USA) for cDNA Synthesis; QuantiNova SYBR Green PCR Kit (Qiagen, Duesseldorf, Nordrhein-Westfalen, Germany) for qPCR. glyceraldehyde-3-phosphate dehydrogenase (GAPDH) was applied as a reference gene. The relative expression of lnc-MAFG-AS1was calculated using the 2^−ΔΔCt^ method. RT-qPCR was performed in triplicate. The primers were constructed according to the previous study (17): lnc-MAFG-AS1: 5′-ATGACGACCCCCAATAAAGGG-3′ (sense); 5′-CACCGACATGGTTACCAGC-3′ (antisense). GAPDH: 5′-GAAGGTGAAGGTCGGAGT-3′ (sense); 5′-GAAGATGGTGACTGGGATTT-3′ (antisense).

### Statistical Analysis

A comparison of lnc-MAFG-AS1 expression was done by using the Wilcoxon matched-pairs signed rank test or the Wilcoxon rank-sum test. The accuracy of lnc-MAFG-AS1 in identifying the different tissues was analyzed using the receiver operator characteristic curve. When evaluating the correlation between lnc-MAFG-AS1 and OS, the patients were classified into lnc-MAFG-AS1 high and low groups on the basis of the median expression of lnc-MAFG-AS1 in the total tumor tissue. The Kaplan–Meier curve for OS was analyzed using the log-rank test. Prognostic factor analysis was performed using univariable and multivariable Cox’s proportional hazard regression. A *p-*value below 0.05 indicated statistical significance. SPSS 24.0 (IBM Corp., Armonk, NY, USA) and GraphPad Prism 7.01 (GraphPad Software Inc., San Diego, CA, USA) were used for analysis and graphing, respectively.

## Results

### Patients’ Features

Analyzed HCC patients showed an age of 57.3 ± 8.9 years; among them, 86.8% were males, and 13.2% were females (Table 1). The BCLC and CNLC stages were assessed according to ECOG PS, Child–Pugh, tumor nodule number, tumor size, etc. It was found that 1.3%, 48.7%, 20.4%, and 29.6% patients belonged to the BCLC stage 0, A, B, and C, respectively; 18.4%, 41.4%, 24.4%, and 15.8% patients belonged to the CNLC stage Ia, Ib, IIa, and IIb, respectively. Detailed information on these patients’ features is given in Table 1.

**Table 1:** Clinical characteristics.

Items	HCC patients (*N* = 152)
Age (years), mean ± SD	57.3 ± 8.9
Gender, no. (%)	
Female	20 (13.2)
Male	132 (86.8)
History of HB, no. (%)	122 (80.3)
History of liver cirrhosis, no. (%)	114 (75.0)
ECOG PS score, no. (%)	
0	107 (70.4)
Score 1	45 (29.6)
Child–Pugh stage, no. (%)	
Stage A	119 (78.3)
Stage B	33 (21.7)
Tumor nodule number, no. (%)	
Unifocal	80 (52.6)
Multifocal	72 (47.4)
Largest tumor size, no. (%)	
<5.0 cm	81 (53.3)
≥5.0 cm	71 (46.7)
BCLC stage, no. (%)	
Stage 0	2 (1.3)
Stage A	74 (48.7)
Stage B	31 (20.4)
Stage C	45 (29.6)
CNLC stage, no. (%)	
Stage Ia	28 (18.4)
Stage Ib	63 (41.4)
Stage IIa	37 (24.4)
Stage IIb	24 (15.8)
ALT (U/L), median (IQR)	29.9 (22.4–45.1)
AST (U/L), median (IQR)	39.3 (27.0–52.8)
ALP (U/L), median (IQR)	94.7 (73.4–154.6)
TBIL (μmol/L), median (IQR)	14.9 (10.7–24.9)
CEA (ng/ml), median (IQR)	4.8 (3.1–7.0)
CA199 (U/ml), median (IQR)	24.7 (14.9–44.7)
AFP (ng/ml), median (IQR)	109.6 (12.0–917.7)

*HCC, hepatocellular carcinoma; SD, standard deviation; HB, hepatitis B; ECOG PS, Eastern Cooperative Oncology Group performance status; BCLC, Barcelona Clinic Liver Cancer; CNLC, China Liver Cancer; ALT, alanine aminotransferase; IQR, interquartile range; AST, aspartate aminotransferase; ALP, alkaline phosphatase; TBIL, total bilirubin; CEA, carcinoembryonic antigen; CA199, carbohydrate antigen 199; AFP, alpha-fetoprotein.*

### Lnc-MAFG-AS1 Dysregulation

**Figure 1:**
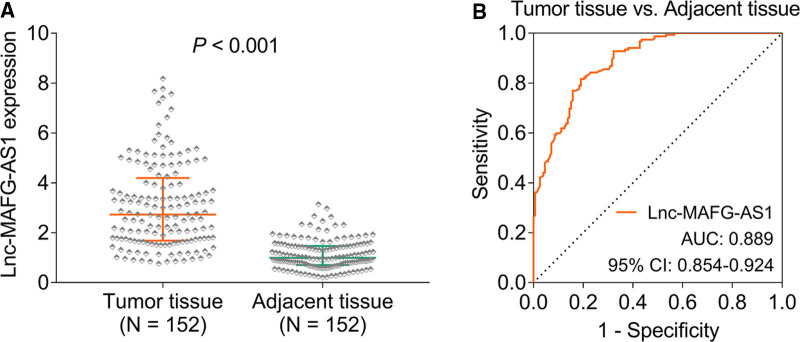
Long non-coding RNAs musculoaponeurotic fibrosarcoma oncogene family, protein G antisense 1 (lnc-MAFG-AS1) expression. Lnc-MAFG-AS1 expression in hepatocellular carcinoma (HCC) tumor tissue and adjacent tissue (**A**). Receiver operator characteristic curve analysis of lnc-MAFG-AS1 expression for distinguishing HCC tumor tissue from adjacent tissue (**B**).

Lnc-MAFG-AS1 was higher in HCC tumor tissue than in adjacent tissue [median (interquartile range): 2.730 (1.685–4.198) vs. 0.990 (0.703–1.468), *p *< 0.001] (Figure 1A). Besides, lnc-MAFG-AS1 distinguished HCC tumor tissue from adjacent tissue with an AUC of 0.889 [95% confidence interval (CI): 0.854–0.924] (Figure 1B).

### Relationship of lnc-MAFG-AS1 with Patients’ Features

In terms of disease conditions and tumor properties, lnc-MAFG-AS1 expression was related to multifocal nodules (*p *< 0.001), increased BCLC stage (*p *= 0.018), and elevated CNLC stage (*p *= 0.008) (Table 2). From the perspective of liver function indexes and tumor markers, lnc-MAFG-AS1 expression was associated with an abnormal alpha-fetoprotein (AFP) level (*p *= 0.004) (Table 3). However, lnc-MAFG-AS1 was not linked with other disease conditions, tumor properties, liver function indexes, or tumor markers in HCC patients.

**Table 2:** Correlation of lnc-MAFG-AS1 expression with patients’ characteristics.

Items	Lnc-MAFG-AS1 expression Median (IQR)	*p*-Value
Age		0.657
<60 years	2.715 (1.630–3.928)	
≥60 years	2.900 (1.758–4.370)	
Gender		0.685
Female	3.040 (1.310–4.798)	
Male	2.730 (1.685–3.945)	
History of HB		0.781
No	2.960 (1.160–4.753)	
Yes	2.730 (1.730–3.965)	
History of liver cirrhosis		0.540
No	2.600 (1.565–3.413)	
Yes	2.750 (1.695–4.328)	
ECOG PS score		0.131
Score 0	2.640 (1.640–3.930)	
Score 1	3.150 (1.850–4.935)	
Child–Pugh stage		0.724
Stage A	2.820 (1.700–3.950)	
Stage B	2.220 (1.545–5.150)	
Tumor nodule number		<0.001
Unifocal	2.150 (1.513–3.300)	
Multifocal	3.380 (2.108–4.895)	
Largest tumor size		0.056
<5.0 cm	2.540 (1.635–3.560)	
≥5.0 cm	3.210 (1.800–4.960)	
BCLC stage		0.018
Stage 0/A	2.275 (1.513–3.583)	
Stage B/C	3.135 (1.915–4.883)	
CNLC stage		0.008
Stage I	2.270 (1.520–3.600)	
Stage II	3.360 (2.025–4.990)	

*Lnc-MAFG-AS1, long non-coding RNAs musculoaponeurotic fibrosarcoma oncogene family, protein G antisense 1; IQR, interquartile range; HB, hepatitis B; ECOG PS, Eastern Cooperative Oncology Group performance status; BCLC, Barcelona Clinic Liver Cancer; CNLC, China Liver Cancer.*

**Table 3:** Correlation of lnc-MAFG-AS1 expression with liver function indexes and tumor markers.

Items	Lnc-MAFG-AS1 expression Median (IQR)	*p*-Value
ALT		0.793
Normal (<40 U/L)	2.610 (1.760–3.950)	
Abnormal (≥40 U/L)	3.240 (1.510–4.755)	
AST		0.081
Normal (<40 U/L)	2.550 (1.665–3.410)	
Abnormal (≥40 U/L)	3.245 (1.725–4.878)	
ALP		0.938
Normal (<128 U/L)	2.715 (1.765–3.695)	
Abnormal (≥128 U/L)	3.140 (1.173–5.080)	
TBIL		0.683
Normal <19 μmol/L	2.880 (1.780–3.780)	
Abnormal (≥19 μmol/L)	2.220 (1.520–5.060)	
CEA		0.366
Normal (<5 ng/ml)	2.640 (1.750–3.530)	
Abnormal (≥5 ng/ml)	2.800 (1.580–4.850)	
CA199		0.190
Normal (<37 U/ml)	2.720 (1.640–3.680)	
Abnormal (≥37 U/ml)	3.030 (1.890–4.910)	
AFP		0.004
Normal (<25 ng/ml)	2.140 (1.633–3.270)	
Abnormal (≥25 ng/ml)	3.220 (1.865–4.865)	

*Lnc-MAFG-AS1, long non-coding RNAs musculoaponeurotic fibrosarcoma oncogene family, protein G antisense 1; IQR, interquartile range; ALT, alanine aminotransferase; AST, aspartate aminotransferase; ALP, alkaline phosphatase; TBIL, total bilirubin; CEA, carcinoembryonic antigen; CA199, carbohydrate antigen 199; AFP, alpha-fetoprotein.*

### Linkage of lnc-MAFG-AS1 with Prognosis

**Figure 2:**
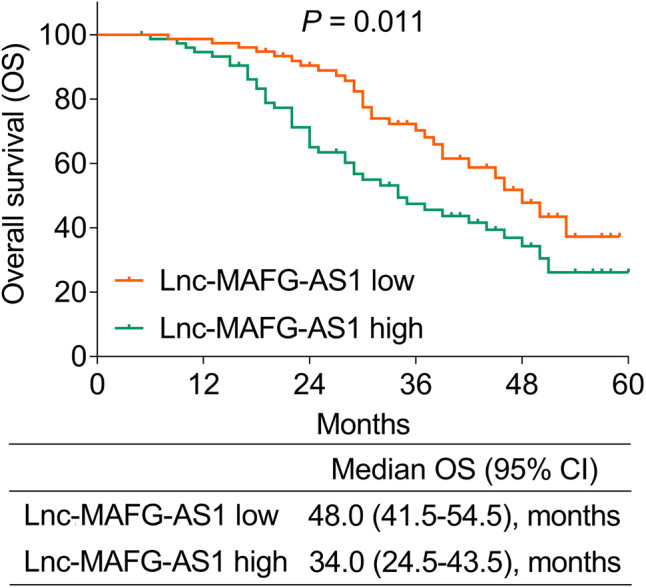
Correlation of lnc-MAFG-AS1 expression with overall survival (OS).

During a median follow-up of 30 months (range: 5–60 months) in this study, unfortunately 71 (46.7%) patients died. It was then observed that patients with a high expression of lnc-MAFG-AS1 exhibited worse OS than those with a low expression of lnc-MAFG-AS1 [median (95% CI): 34.0 (24.5–43.5) vs. 48.0 (41.5–54.5) months] (*p *= 0.011) (Figure 2). In detail, the 1-, 2-, 3-, and 4-year OS rates of patients with low lnc-MAFG-AS1 were 97.4%, 90.4%, 70.3%, and 47.8%, respectively. The 1-, 2-, 3-, and 4-year OS rates of those with high lnc-MAFG-AS1 were 94.6%, 65.0%, 47.5%, and 34.3%, respectively.

**Figure 3:**
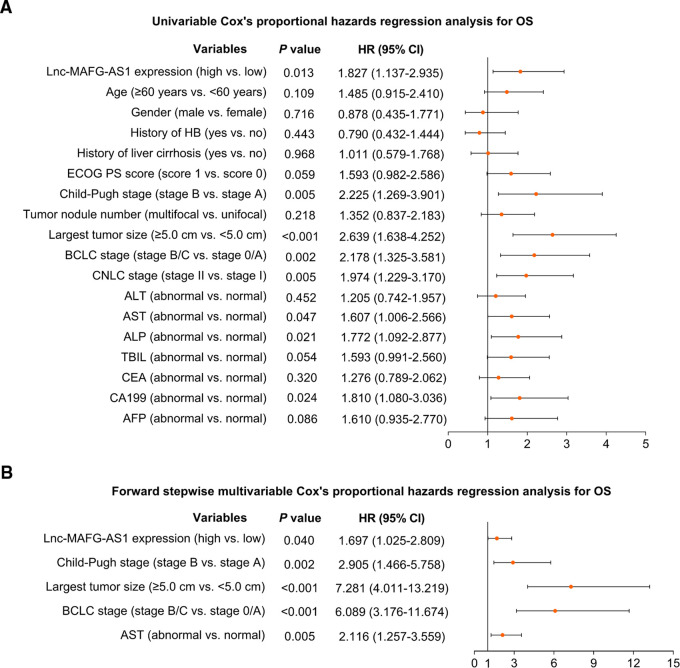
Prognostic factors relating to OS. Univariate (**A**) and multivariate (**B**) Cox’s proportional hazards regression analysis of prognostic factors relating to OS.

In addition, univariate Cox’s analysis found that lnc-MAFG-AS1 expression (high vs. low) correlated with unfavorable OS [hazard ratio (HR) = 1.827, *p *= 0.013] (Figure 3A). Subsequent multivariate Cox’s analysis identified that lnc-MAFG-AS1 expression (high vs. low) related to a less-prolonged OS (HR = 1.697, *p *= 0.040) (Figure 3B).

**Figure 4:**
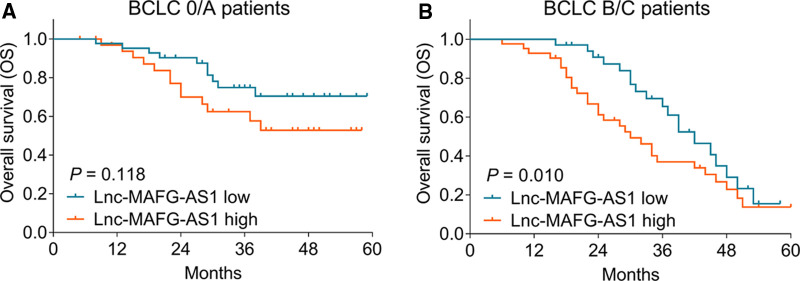
Subgroup analysis. Correlation of lnc-MAFG-AS1 expression with OS in the Barcelona Clinic Liver Cancer (BCLC) 0/A subgroup (**A**) and BCLC B/C subgroup (**B**).

Furthermore, subgroup analysis was conducted, which disclosed that high lnc-MAFG-AS1 was not related to OS in BCLC 0/A patients (*p *= 0.118) (Figure 4A) but was linked with worse OS in BCLC B/C patients (*p *= 0.010) (Figure 4B).

## Discussion

Many lncRNAs have been discovered to be ontogenetic, such as lncRNA NEAT1, lncRNA GAS5, and lncRNA MALAT1 (18–24). With regard to lnc-MAFG-AS1, it exhibits tumorigenesis by regulating the miR-574/SOD2 axis in breast cancer (9); it also reverses miR-34a maturation to induce glioblastoma growth (10); besides, it accelerates pancreatic cancer progression by binding miR-3196 to outburst NFIX (25). It is also worth noting that lnc-MAFG-AS1 serves as a potential biomarker for prognostication in several cancers (17, 26–28). For example, lnc-MAFG-AS1 correlates with invasive depth, tumor, node, metastasis (TNM) stage and unsatisfied disease-free survival (DFS), and OS in colorectal cancer patients (17); it is also related to diffuse type, extensive invasion depth, frequent lymph node metastasis, and distant metastasis and predicts worse OS in gastric cancer patients (26).

Apart from the carcinogenic role of lnc-MAFG-AS1 in other cancers, it is also closely involved in HCC pathogenesis and treatment response (13–15, 29). For instance, lnc-MAFG-AS1 enhances HCC cell proliferation, migration, and invasion, which is hampered by miR-6852 (13), which also facilitates these malignant behaviors of HCC via miR-3196-mediated OTX1 (14). Then, silencing lnc-MAFG-AS1 expression is found to impede the tumor progression of HCC both *in vitro* and *in vivo* by interacting with NM IIA subunits (MYH9, MYL12B, and MYL6) (29). More inspiringly, lnc-MAFG-AS1 is engaged in the drug resistance of HCC through the miR-3196/STRN4 axis (15). However, the clinical involvement of lnc-MAFG-AS1 in HCC patients remains obscure. The current study observed that lnc-MAFG-AS1 was upregulated in HCC tissue compared with that in adjacent tissue. This may result from the tumorigenesis role of lnc-MAFG-AS1 to promote HCC development. Therefore, it is overexpressed in tumor tissue and not in adjacent non-tumor tissue (9, 10, 13, 14).

In addition, the present study also discovered that lnc-MAFG-AS1 expression was related to multifocal nodules, increased BCLC stage, elevated CNLC stage, and abnormal AFP level in HCC patients. The possible explanations are listed: First, lnc-MAFG-AS1 promotes HCC migration and invasion in multiple ways, such as miR-6852, miR-3196-mediated OTX1, MYH9, MYL12B, and MYL6, to facilitate the development of multifocal nodules (13, 14). Second, lnc-MAFG-AS1 enhances tumor growth, mobility, and EMT in multiple ways mentioned above, and, therefore, correlates with advanced tumor stages (BCLC stage and CNLC stage) (13–15, 29). Third, lnc-MAFG-AS1 induces HCC progression to secrete more AFP. Therefore, it is related to an abnormal AFP level (13–15, 29).

It was also uncovered that lnc-MAFG-AS1 related to unfavorable OS in HCC patients, which was validated by using K–M curve analysis, univariate Cox’s analysis, and multivariate Cox’s analysis. The possible explanations are: First, lnc-MAFG-AS1 relates to multifocal nodules, increased BCLC stage, elevated CNLC stage, and abnormal AFP level in HCC patients, which are indirectly related to deteriorative prognosis. Second, lnc-MAFG-AS1 regulates the drug resistance of HCC, which then affects the preoperative and postoperative treatment response, or the response to therapy after relapse, to directly relate to HCC prognosis (15).

Several limitations could be mentioned in the study. First, because the enrolled HCC patients were mostly not local patients, the treatment they received during the follow-up period and a detailed DFS information could not be obtained, leading to a lack of proper analysis. Second, only resectable HCC patients were included in this study. Therefore, the related findings might not be suitable for unresectable HCC patients.

In conclusion, lnc-MAFG-AS1 relates to multifocal nodules, increased BCLC stage, elevated CNLC stage, and abnormal AFP level and predicts pejorative prognosis in HCC patients. These findings may help prognostic risk stratification of HCC, while further validations are needed.

## Data Availability

The original contributions presented in the study are included in the article/supplementary material; further inquiries can be directed to the corresponding author/s.
